# Supplementary material for: A two-step mechanism for sugar translocation
Source: Nat Struct Mol Biol. 2026 Apr 8;33(4):652–63. doi: 10.1038/s41594-026-01784-w (PMC13095657; doi:10.1038/s41594-026-01784-w)
Supplement: Supplementary file 1 — Supplementary Tables 1–4 and Video legend. [file 41594_2026_1784_MOESM1_ESM.pdf]

nature structural & molecular biology

Supplementary information https://doi.org/10.1038/s41594-026-01784-w

A two-step mechanism for sugar translocation

In the format provided by the authors and unedited

The PDF file includes:

Supplementary Tables 1-4

Supplementary Table 1. ^{1}H and STD intensities of D-xylose and D- glucose in the presence of the indicated transporters.

D-xylose		^{1}H intensity	STD intensity
	XylE wild-type	7,368,907,392	2,689,704,992
	XylE V35C/E302C reduced	8,589,200,704	2,101,279,136
	XylE V35C/E302C oxidized	8,484,326,912	917,635,040
	XylE G58W/L315W	9,470,401,280	54,247,744
	XylE Q175I/L297F	10,426,632,320	1,962,936,512
	XylE wild-type in brain fraction 7 lipid	6,347,163,437	803,366,628

D- glucose		^{1}H intensity	STD intensity
	XylE wild-type	16,814,787,840	454,581,456
	XylE Q175I/L297F	12,288,783,936	812,448,200
	XylE wild-type in brain fraction 7 lipid	7,382,777,782	184,619,244
	*Pf*HT1 wild-type	6,759,996,032	1,478,832,176
	*Pf*HT1 K51Q	306,116,139,492	45,517,287,442
	*Pf*HT1 Q305A	386,307,122,412	19,654,550,719
	*Pf*HT1 Q306A	334,932,163,348	11,510,797,008
	*Pf*HT1 N311A	323,740,085,071	19,915,283,276
	*Pf*HT1 V314Y	304,326,440,576	15,279,131,026
	*Pf*HT1 S315A	324,588,605,045	19,236,218,324
	*Pf*HT1 N316A	294,430,422,302	24,303,852,591
	*Pf*HT1 N341A	265,952,238,170	9,440,386,998
	*Pf*HT1 A404E	307,892,754,501	33,806,231,492
	*Pf*HT1 W412A	275,969,167,220	40,941,978,471

Supplementary Table 2. ^{1}H and STD intensities of D-fructose and 2,5-AHM in the presence of the indicated transporters.

D-fructose		^{1}H intensity	STD intensity
	*Pf*HT1 wild-type	1,710,198,144	58,598,512
	rGLUT5 wild-type	22,265,087,014	4,225,686,903
	rGLUT5 N293A	384,855,228	14,022,467
	rGLUT5 Y296F	528,582,584	65,219,258
	rGLUT5 Y297A	469,904,558	48,828,448
	rGLUT5 Y298A	505,590,951	17,273,308
	rGLUT5 Q301R	326,386,724	37,988,430

2,5-AHM		^{1}H intensity	STD intensity
	*Pf*HT1 wild-type	465,987,352,376	87,666,424,364
	*Pf*HT1 K51Q	622,385,933,048	79,362,577,837
	*Pf*HT1 Q305A	583,793,014,240	112,065,470,856
	*Pf*HT1 Q306A	369,861,279,760	14,675,283,930
	*Pf*HT1 N311A	631,935,149,592	20,321,037,745
	*Pf*HT1 V314Y	468,204,296,528	12,089,069,273
	*Pf*HT1 S315A	674,823,156,208	50,588,060,025
	*Pf*HT1 N316A	325,677,108,984	27,657,447,976
	*Pf*HT1 N341A	350,417,560,004	8,931,159,032
	*Pf*HT1 A404E	353,154,730,440	118,894,850,214
	*Pf*HT1 W412A	482,838,614,104	27,953,615,378

Supplementary Table 3. Clusters of D-xylose position. The table reports the number of total and per-replicate instances in which D-xylose is found in a given cluster; only clusters occupied for 0.5% of total simulation time or more are shown. The first cluster represents the crystal-like sugar pose.

Cluster nr.	Tot. Instances	% of tot. sim.time	Repeat 1 instances (%)	Repeat 2 instances (%)	Repeat 3 instances (%)	Repeat 4 instances (%)	Repeat 5 instances (%)
1	1771	35.38	635 (63.5%)	94 (9.4%)	146 (14.6%)	829 (82.9%)	67 (6.67%)
2	151	3.01	6 (0.6%)	101 (10.1%)	23 (2.3%)	0 (0%)	21 (2.1%)
3	111	2.21	0 (0%)	0 (0%)	0 (0%)	111 (11.1%)	0 (0%)

Supplementary Table 4. Clusters of glucose position. The table reports the number of total and per-replicate instances in which glucose is found in a given cluster; only clusters occupied for 1% of total simulation time or more are shown. The first cluster represents the crystal-like sugar pose.

Cluster nr.	Tot. Instances	% of tot. sim.time	Repeat 1 instances (%)	Repeat 2 instances (%)	Repeat 3 instances (%)	Repeat 4 instances (%)	Repeat 5 instances (%)
1	2355	47.05	461 (46.1%)	701 (70%)	636 (63.5%)	147 (14.7%)	410 (41%)
2	144	2.88	5 (0.5%)	0 (0%)	69 (6.9%)	0 (0%)	70 (7%)
3	98	1.96	97 (9.7%)	0 (0%)	0 (0%)	0 (0%)	1 (0.1%)
4	83	1.66	0 (0%)	0 (0%)	13 (1.3%)	4 (0.4%)	66 (6.6%)
5	72	1.44	58 (5.8%)	9 (0.9%)	0 (0%)	3 (0.3%)	2 (0.2%)
9	66	1.32	0 (0%)	0 (0%)	66 (6.6%)	0 (0%)	0 (0%)
